# Adherence with epinephrine autoinjector prescriptions in primary care

**DOI:** 10.1186/s13223-017-0218-5

**Published:** 2017-11-10

**Authors:** Elissa M. Abrams, Alexander G. Singer, Lisa Lix, Alan Katz, Marina Yogendran, F. Estelle R. Simons

**Affiliations:** 1 Department of Pediatrics, Section of Allergy and Clinical Immunology, University of Manitoba, FE125-685 William Avenue, Winnipeg, MB R3E 0Z2 Canada; 2 Department of Family Medicine, University of Manitoba, Winnipeg, Canada; 3 Department of Community Health Sciences, University of Manitoba, Winnipeg, Canada; 4 Department of Family Medicine and Community Health Sciences, University of Manitoba, Winnipeg, Canada; 5 Manitoba Centre for Health Policy, University of Manitoba, Winnipeg, Canada

**Keywords:** Anaphylaxis, Medication adherence, Epinephrine autoinjector

## Abstract

**Background:**

The aim of this study was to estimate primary adherence for epinephrine autoinjector (EA) prescriptions in primary care practices in Manitoba, Canada.

**Methods:**

A retrospective analysis of electronic medical record and administrative data was performed to determine primary adherence, defined as dispensation of a new EA prescription within 90 days of the date the prescription was written. Multivariable logistic regression models were used to test predictors of filling an EA prescription.

**Results:**

Of 1212 EA prescriptions written between 2012 and 2014, only 69.9% (N = 847) were filled. An increased number of prescriptions for non-EA mediations was associated with an increased odds ratio of not filling an EA prescription.

**Interpretation:**

This is the first study in Canada to examine adherence for EA prescriptions. The non-adherence rate identified is higher than rates previously reported in the literature, and indicates that many EA prescriptions for adults seen in primary care may never be filled. It also suggests that prescriptions of EAs for all patients at risk of anaphylaxis in community settings should consistently be accompanied by concise information about the importance of having the EA prescription filled and having the EA readily available.

## Main text

### Introduction

There is international consensus that patients at risk for anaphylaxis in the community setting have an epinephrine autoinjector (EA) available at all times [1, 2]. Epinephrine’s beneficial mechanisms of action include reduction in mast cell mediator release, vasoconstriction, inotropic and chronotropic effects and bronchodilation [1, 2]. It can prevent and reduce symptoms and signs in all body organ systems involved in anaphylaxis (skin, upper and lower respiratory tract, gastrointestinal tract, and vasculature) [1, 2]. Injectable epinephrine is the only life-saving intervention available for anaphylaxis [1], and delayed or lack of epinephrine use is a risk factor for anaphylaxis morbidity and mortality [3, 4]. As death from anaphylaxis can occur quickly (within minutes), prompt treatment with epinephrine is required [1, 2].

Previous studies have noted that EAs are often under-prescribed and under-utilized. Many patients with a history of anaphylaxis are not prescribed an EA, or do not carry it with them at all times [5, 6]. For example, a survey of 1885 patients with a history of anaphylactic reactions noted that EAs were used in only 27% of these episodes [6]. Of those who didn’t use an EA, reasons included taking an H_1_-antihistamine instead (38%) or having no EA prescription (28%). However, there have been few studies examining rates of EA primary adherence (i.e., the rate of filling a first prescription for an EA).

The objective of this study was to determine EA primary prescription adherence in an adult primary care patient population in Manitoba over a 2-year period.

### Methods

A retrospective analysis was performed on data from the Manitoba Primary Care Research Network (MaPCReN), a repository of de-identified primary care electronic medical record data. At the time of this study, MaPCReN included 44 primary care clinics representing 241 providers caring for over 200,000 patients age 18 years and older. Prescriptions written from April 1st, 2012 to December 31st, 2014 from the MaPCReN database were linked to Manitoba’s Drug Program Information Network (DPIN) data housed at the Manitoba Centre for Healthy Policy for medications that belong to the Anatomical Therapeutic Chemical (ATC) classification corresponding to an epinephrine autoinjector [7]. The DPIN contains data for all prescriptions dispensed by community pharmacies in the province of Manitoba.

The outcome was primary adherence, defined as dispensation (i.e., filling) of a new prescription (i.e., a prescription not dispensed to that patient within the prior 365 days) within 90 days of the date the prescription was written. An exclusion criteria was a hospital admission within 90 days post writing of the prescription.

Multivariable logistic regression analysis was used to test predictors of EA primary adherence, including: age, sex, income quintile, measures of healthcare use (i.e., medical diagnoses, number of hospitalizations, number of primary care visits, number of emergency department visits, number of non-EA prescriptions dispensed), and the Charlson Comorbidity index (a composite medical complexity score that predicts mortality). Analyses were conducted using SAS Version 9.4.

### Results

Table 1 describes the demographic characteristics and adherence information of the patient population. The majority of patients were female (66.2%; N = 802). The most common age group was 18–44 years (47.7%; N = 578) followed by 45–64 years (40.4%; N = 490).

**Table 1:** Sample demographics

Variable	Filled (n, %)	Unfilled (n, %)	OR (95% CI)
Sex			
Male	269 (22.2)	141 (11.6)	Ref
Female	578 (47.7)	224 (18.5)	1.24 (0.95, 1.62)
Age			
18–44	410 (33.8)	168 (13.9)	*1.66 (1.07, 2.58)*
45–64	342 (28.2)	148 (12.2)	1.43 (0.94, 2.18)
65 +	95 (7.8)	49 (4.1)	Ref
Total	874 (69.9)	365 (30.1)	

Study sample with demographic characteristics and their effect on the likelihood of adherence from multivariable logistic regression

Females are compared to males and age groups compared to over 65 year old grouping

Odds ratio (OR) in italic is statistically significant using a nominal α = .05

During the study period, the patient population had 1212 new prescriptions written for an EA. A total of 69.9% (N = 847) of these prescriptions were filled within 90 days. The number of non-EA prescriptions dispensed (6 or more non-EA dispensings, compared to 0–2 non-EA dispensings) was associated with an increased odds that an EA prescription would not be filled (OR = 2.24, 95% CI 1.44–3.47) in multivariate analysis. All other model covariates (age, sex, income quintile, medical diagnoses, number of hospitalizations, number of primary care visits, number of emergency department visits, and the Charlson Comorbidity index) were not statistically significant.

### Discussion

To our knowledge this is the first Canadian study to examine primary adherence for EA prescriptions. It identifies significant non-adherence with a life-saving medication, with only about two-thirds of EA prescriptions being filled. In addition, this study identifies polypharmacy as being significantly associated with EA prescription primary non-adherence. This finding is especially concerning given the results of previous studies identifying that EAs are often not prescribed, even when indicated [6].

Our findings contrast with previous studies, which have reported a higher rate of EA prescription adherence [8, 9]. A retrospective review of electronic medical records at an American military medical centre noted that of 881 self-injectable epinephrine prescriptions for 769 patients, 82% of patients filled at least one prescription, with increased adherence linked to increased age (> 55 years, P < 0.009) [8]. A recent retrospective cohort study of patients with anaphylaxis in a large US Health Maintenance Organization reported that 95.9% of prescriptions were dispensed, independent of copayment amount [9]. Two studies have also found high EA prescription adherence based on self-report [6, 10]. Specifically, an anonymous survey of 120 families seen at a US pediatric allergy clinic found that 94% of those who were prescribed an EA reported filling the prescription [10]. An survey of 1885 individuals who had survived anaphylaxis or cared for someone who survived anaphylaxis found that EA prescription adherence was about 99%, based on self-report [6]. However, this study also identified that the majority (73%) of patients prescribed an epinephrine autoinjector were nonusers. Reasons contributing to this included using an H_1_-antihistamine (38%) or no prescription for epinephrine (28%).

We anticipated that our study would identify socioeconomic status as a variable associated with EA primary adherence. Other studies have found health disparities in prescription patterns of EAs [11, 12]. For example, a population-based survey of school children noted that children from upper-middle and high-income homes were significantly more likely to have an epinephrine prescription (OR = 8.35, 95% CI 2.72–25.61) [11]. As a result, we anticipated that patients with low income would have lower rates of primary adherence due to poorer access to health care and factors such as affordability of medications. However, our study found no association between EA prescription adherence and socioeconomic status in this adult population. Of note, we did not have individual socioeconomic status, rather, we used income quintile as an indicator of ecological socioeconomic status. In addition, those in the lowest quintile were more likely to be eligible for social assistance which may partially explain this finding.

We found that the only variable associated with EA prescription non-adherence was the number of non-EA prescriptions, suggesting that more diligence is required about education on the importance of EA prescription adherence.

A limitation of our retrospective analysis is that our study population is from a single Canadian province, and may not be representative of other primary care populations. We did not capture EAs prescribed by allergists, emergency medicine specialists, internists, pediatricians or other specialists from whom primary adherence rates may vary. Our sample was comprised only of adults; primary adherence rates may be different in populations of children and youth. An exclusion criteria of the dataset was a hospital admission within 90 days of the prescription, and hence it is possible that some patients with unfilled prescriptions were admitted to hospital with anaphylaxis and not captured in the database. Finally, although the Charlson index has been validated in a wide variety of populations, it has not been validated in an outpatient population with allergic disease.

Despite these limitations, this study provides important insights about the lack of prescription primary adherence in the Canadian population, and indicates that many EA prescriptions for adults seen in primary care may never be filled. Further studies are needed to determine the reasons for EA prescription non-adherence.

It also suggests that EA prescriptions for all patients at risk of anaphylaxis in community settings should consistently be accompanied by concise information about the importance of having the EA prescription filled and having the EA readily available. A brief presentation and a summary card given to time-challenged primary care providers has been shown to significantly improve recall of important information on anaphylaxis and EAs [13]. Along with their primary care colleagues, allergists share the role of educating patients about the importance of EA prescription adherence and could potentially work together with them to develop and validate a practical anaphylaxis/EA education tool.

## Abbreviations

DPIN: Manitoba’s Drug Program Information Network
EA: epinephrine autoinjector
MaPCReN: Manitoba Primary Care Research Network
